# Influence of Nursing Time and Staffing on Medication Errors: A Cross-Sectional Analysis of Administrative Data

**DOI:** 10.3390/nursrep15010012

**Published:** 2025-01-05

**Authors:** Mutsuko Moriwaki, Michiko Tanaka, Masayuki Kakehashi, Masato Koizumi, Hiromasa Horiguchi, Kenshi Hayashida

**Affiliations:** 1 Quality Management Center, Institute of Science Tokyo Hospital, 1-5-45 Yushima, Bunkyo-ku, Tokyo 113-8519, Japan; 2 Department of Clinical Data Management and Research, Clinical Research Center, National Hospital Organization Headquarters, Tokyo 152-8621, Japan; 3 Department of Nursing, Daiichi University of Pharmacy, Fukuoka 815-8511, Japan; 4 Graduate School of Biomedical and Health Sciences, Hiroshima University, Hiroshima 734-8553, Japan; 5 Department of Medical Informatics and Management, University Hospital, University of Occupational and Environmental Health, Kitakyusyu 807-8555, Japan

**Keywords:** nursing time, medication errors, staffing, patient safety, ward activity

## Abstract

**Background**: Medication errors cause adverse events; however, studies have yet to examine medication errors related to nursing hours while considering ward characteristics in Japan. Purpose: This study investigated medication errors caused by nurses to quantitatively assess ward activity as busyness in nursing duties. **Methods**: This study considered patients hospitalized in the general wards of 10 National Hospital Organization institutions between April 2019 and March 2020. The study data were obtained from the Diagnosis Procedure Combination system, incident report system, and reports on nurse staffing and work hours. Data for 27,629 ward days with 88,475 patients were analyzed. Multivariate analysis was performed to determine the impact of factors on medication errors. **Results**: The mean patient age was 71.43 years (SD = 15.08). The medication error rate in nursing wards was 13.71%. The mean nursing time per patient during day shift was 1.95 h (SD = 0.58) in the non-medication error group and 2.06 h (SD = 0.58) in the medication error group (*p* < 0.01). The nursing time per patient in the medication error group compared to that in the non-medication error group had an odds ratio of 1.31 (*p* < 0.01) during day shifts. **Conclusions/Implications for practice**: Contrary to evidence, the results showed that medication errors caused by nurses related to increased nurse time with patients during day shifts. Further investigation is needed on the relationship of busyness with nursing duties to ensure an adequate nurse–patient ratio, nursing time, and improved patient safety.

## 1. Introduction

Medication errors are “any preventable event that may cause or lead to inappropriate medication use or patient harm while the medication is controlled by the health-care professional, patient, or consumer” [1]. A study conducted in 2017 reported that medication errors caused over 250,000 deaths annually and were the nation’s third leading cause of death [2]. Possible causes of medication errors are multiple competing demands, which require nurses to switch attention between overlapping tasks, and a shortage of nurses, which affects nurse staffing.

The assignment of nurses in medical institutions in Japan must comply with the standards stipulated by the medical service fee system and the optimal standards stipulated by the Medical Service Act. In 2006, the patient-to-nurse ratio system was introduced, linking the number of nurses to medical service fees. The nurse staffing standards stipulated in the reimbursement system are defined in the basic hospitalization charges [3]. The patient-to-nurse ratios in general wards are 7:1, 10:1, 13:1, or 15:1, and medical institutions must adopt one of these levels. For example, “7:1” means that at least one nursing staff member provides care to seven hospitalized patients per day, indicating that the average daily patient-to-nurse ratio is 7:1. Specifically, a patient-to-nurse ratio of approximately 5 to 6:1 can be achieved in the day shift. A nurse can be assigned at approximately 14:1 on the night shift. Thus, nurse staffing can be adjusted flexibly according to patient conditions and the workload of the ward. However, from a managerial standpoint, it is difficult for medical institutions to assign nurses beyond the basic inpatient fee stipulations, and it is unclear whether the necessary nurses are appropriately assigned to hospital wards. On the other hand, the nurses’ workload in Japan is increasing due to the aging of hospitalized patients, shorter hospital stays, and more sophisticated medical care [4]. However, staffing does not necessarily reflect the increase in the number of admissions and discharges, procedures, and other nursing activities. In many cases, nursing assignments are determined by the number of patients and nurses. As a result, nurses often cannot adequately monitor patients to ensure their safety.

Strong evidence exists of the relationship between nurse staffing and patient outcomes [5,6,7]. Previous research has revealed that allowing nurses to spend more time at patients’ bedsides reduces hospital infection rates, rehospitalization rates, and patient mortality while improving glycemic control [8,9,10,11,12]. Additionally, Lasater et al. reported that improving the nurse–patient ratio decreases mortality, the length of hospital stays, rehospitalization rates, and costs [8]. Therefore, appropriate nurse staffing reduces medication errors and improves patient outcomes.

Nurse managers may control daily nurse staffing based on patients’ conditions, nurses’ experience, and the ward workload. However, the adequacy of methods that measure nurse staffing and workload is unclear, and there is no consensus regarding their appropriateness [13,14,15,16].

Furthermore, no studies have examined the medication errors related to nurse staffing or nursing hours, including ward activity, as a characteristic of wards in Japan. Therefore, this study aimed to evaluate the association between nursing hours per patient and the incidence of drug-related adverse events caused by nurses in a broad range of practice settings. The study focused on medication errors caused by nurses; therefore, falls, which are strongly affected by patient characteristics and status, were excluded.

## 2. Materials and Methods

### 2.1. Study Population

We conducted a retrospective study using data from an acute care hospital. The study population included patients from 10 National Hospital Organization (NHO) facilities in Japan. The NHO was established to manage national hospitals in Japan and included 140 hospitals as of March 2020. All participants were in general wards between April 2019 and March 2020. Those from critical care wards, such as intensive care units (ICUs) and high care units, obstetrics/gynecology, and pediatrics wards were excluded. The number of target patients, medication errors, and working hours of nurses were aggregated and consolidated on a ward-day basis. In total, 27,629 ward days (88,475 cases, counting a hospitalization as one) were included in the analysis.

Additionally, wards established for a limited period, incidents unrelated to medication, incidents not involving nurses, and ward data with outlier nursing time per patient during day or night shifts were excluded. Data without ward codes or dates entered in the DPC data, Form 9, or the incident reporting system were treated as missing values.

### 2.2. Data Sources

Data were collected from the Diagnosis Procedure Combination (DPC) system, work record notification system (Format 9), and incident report system (limited to medication errors caused by nurses) databases at each NHO.

The DPC system is a case-mix patient classification system for acute inpatient hospitals in Japan. It has been utilized to report medical information in the Japanese Labor and Welfare. As of 2021, 1757 medical institutions and 54.4% of acute hospital beds in Japan were covered by this system. In addition, the DPC database includes the Severity of a Patient’s Condition and the Extent of a Patient’s Need for Medical/Nursing Care (SCNMN) database. The DPC database is an index for measuring the amount of nursing care required for inpatients. The index consists of Item A (seven items), evaluating the status of monitoring and treatment; Item B (seven items), evaluating the activities of daily living (ADL) and other conditions of patients; and Item C (seven items), evaluating the implementation status of medical care related to surgery and emergency medicine [17,18].

Format 9 is a record of individual nursing time, excluding overtime. It has a national unification format to report nurse staffing and nursing time as references for basic hospitalization fees, which are claimed payments by medical facilities to the government.

Adverse event reports, required under the Ordinance for Enforcement of the Medical Care Act, are a part of the hospital’s incident report system and are mandatory for universities and national hospitals. Events of medication errors were extracted from the accumulated incident cases.

### 2.3. Measures and Outcomes

This study defined ward days on which medication errors occurred.

In this study, medication errors included the following:

1. Misidentified patients for nursing practices (treatment, medication, examination, and serving meals), e.g., treatment for patient A was provided to patient B.
2. Misidentified nursing practices (treatment, medication, examination, and serving meals), e.g., Patient A was provided treatment B instead of prescribed treatment C.
3. Misidentification of medication by nurses included misidentifying patients for medication and the misadministration of medication (dosing errors and dosing method errors).
4. Medication errors caused by nurses. We excluded medication errors associated with the patient, consumer, and other healthcare providers, such as physicians, pharmacologists, and nursing assistants, and outside-hospital care (while staying outside the hospital) based on case content.

The primary outcomes in this study adhered to ward-day levels with nursing duration (day or night shift). The daily working hours of nurses during the day (8 h) and night (16 h) were recorded in Format 9 in each ward. The nursing time per patient during the day/night was calculated by adding the values (daily hours) divided by the number of patients.

### 2.4. Patient Variables

For variables indicating the workload on ward days, we obtained data on the sex and age distributions (sociodemographic distributions) of patients hospitalized in the ward on that day, the clinical condition of patients, the status of nursing care, and the patient’s physical functions. This study used values obtained by dividing the number of applicable patients by the total number of patients in the ward on that day.

Age and sex were used to assess sociodemographic status. Regarding the clinical condition of patients, we obtained data on the use of psychotropic medications, hypertension, osteoporosis, anemia, comorbidities during hospitalization, and emergency hospitalizations. Comorbidity scores were calculated using the Quan version [19] of the Charlson Comorbidity Index [20]. The daily working hours of each nurse during the day (8 h) and night (16 h) were recorded in Format 9 for each ward. The nursing time per patient was calculated by summing the values (hours) for each day and dividing them by the number of patients (Nursing time per patient = Total working hours of nurses/Number of patients).

The SCNMN index A, B, and C indicate patients’ conditions, nursing care status, and ward workload.

The occurrence of medication errors is considered to be influenced by specialization or average-case complexity. These factors require capturing more detailed information, such as patient conditions and ward workload, rather than relying solely on diagnoses or ward specializations. Therefore, the analysis was conducted using SCNMN and CCI score data.

### 2.5. Statistical Analyses

Ward days with medication errors were considered the medication error group, while those without medication errors were considered the non-medication error group. Descriptive statistics were performed to distribute the number of patients per ward and nursing time during the day/night. Group variables were compared using the Mann–Whitney U test and the chi-squared test. Stepwise regression analysis used the medication error group as the dependent variable. IBM SPSS Statistics for Windows, version 29 (IBM Corp., Armonk, NY, USA) was used for statistical analysis (statistical significance was set at *p* < 0.01 or 0.05).

### 2.6. Ethical Considerations

This study was approved by the Ethical Review Board of the Graduate School of Medicine, Institute of Science Tokyo (approval number: M2018-284, date of approval: 19 April 2019). The requirement for informed consent was waived by the Ethical Review Board because research participants had earlier provided comprehensive consent regarding the use of medical administrative data. All personal information was excluded, and de-identified data were sent to the researchers for secondary use.

## 3. Results

**Figure 1:**
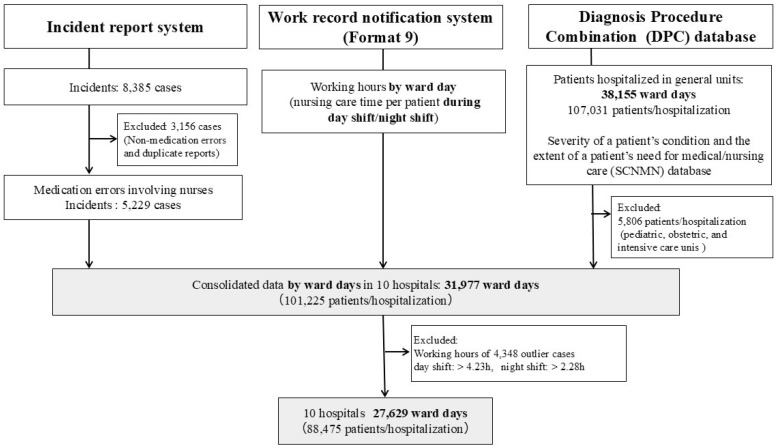
Flowchart of sample section.

We collected medication error data for 31,977 ward days in 10 hospitals on a day-and-ward basis. Outliers with nursing time per patient > 4.23 h in the day shift and >2.28 h in the night shift and a ward established for a limited time were excluded from the analyses, resulting in 27,629 ward days and 88,475 patients (Figure 1). These 10 hospitals were included in the analysis as permission was obtained for the provision of DPC data, incident reports, and Form 9 information.

Medication errors were observed on 3789 ward days and not 23,840 ward days. The mean age of patients was 71.43 years (SD = 15.08), with men accounting for 54.8%. The mean nursing time per patient was 1.96 h (SD = 0.58) in the day shift and 1.46 h (SD = 0.24) in the night shift (Table 1). The medication error rate in the wards was 13.71% (Supplementary Table S1). The average number of patients per ward per day was 41.6 (SD = 6.4) (Supplementary Table S2).

The mean nursing time per patient in the day shift was 1.95 h (SD = 0.58) in the non-medication error group and 2.06 h (SD = 0.58) in the medication error group (*p* < 0.01). The mean nursing time per patient in the night shift was 1.46 h (SD = 0.24) in both groups. The proportion of patients aged ≥ 65 years was higher in the medication error group (*p* < 0.01) (Table 2). A significant difference was observed in ward characteristics for medication error events in sedative-iv hypnotics, psychotropic, and injection use in patients, characteristics of monitoring respiratory care, syringe driver management, radiation therapy, and intensive surgical treatment such as percutaneous endovascular treatment, percutaneous myocardial ablation, invasive gastrointestinal treatment, and eye surgery (Table 3).

Logistic regression analysis showed that compared to the non-medication error group, the medication error group had an odds ratio (OR) of 1.31 (*p* < 0.01) for nursing time per patient in the day shift, which indicates that an increase in nursing time per patient in the day increased medication errors. Hospital characteristics also impacted medication errors (OR = 1.51–3.52) (Table 4).

## 4. Discussion

The results showed that increased nursing time in the day shift did not reduce medication errors. Previous studies revealed that increasing nursing time, nurse–patient ratio, and nursing skill levels improve patient outcomes [8,11,21,22,23]. The present finding that increased nursing time induces medication errors contradicts previous studies’ findings. However, considering the impact of busyness on the nursing workload on the day shift, nurse staffing is critical to protect patient safety. Furthermore, nursing hours per patient in the day shift were higher, and medication errors were more frequent on weekdays, which burdened nursing duties more. The increased medication errors on weekdays may be due to nursing personnel allocated to operational activities such as surgeries, examinations, and medication administration. Medication errors decreased with longer nursing hours in the night shifts, and an increase of one hour in nursing time per patient reduced medication errors by approximately 55%. The quality of nursing time differed between day and night shifts. The results suggest that in places like Japan, where nursing duties are diverse and include not only patient observation and medication but also caregiving, an increase in nursing time per patient may simply indicate an increase in the nurses’ workload. In such cases, it is possible that the staffing arrangement does not ensure patient safety. Previous studies indicate that the daily census and activities are positively associated with medication administration errors [24], suggesting that an increase in workload may influence the rise in medication incidents. In contrast, during the night shift, it can be assumed that the workload is extremely low, allowing for a nursing arrangement that ensures patient safety. In such cases, an increase in nursing time is thought to influence the reduction in medication errors. However, additional investigation is necessary to support this reasoning.

Nurse staffing issues are vital worldwide since the nurse–patient ratio impacts patient mortality, morbidity, and high turnover [5]. Nursing staff increasingly favor positive patient outcomes, although it is difficult to draw robust conclusions between nurse staffing and outcomes in Japanese hospitals [23]. A scoping review revealed that nurses’ perceived positive adequacy of staffing was related to positive outcomes in patients, nurses, and organizations [25]. Registered nurses spend more time providing indirect care than direct patient care, and interruption consumes 17.4% of their time [26]. A time and motion study revealed that nurses spent approximately 10% of their time in non-nursing delegable activities, and less than one-third of their work time was spent with patients [27,28]. Improving the work environment in hospitals can reduce medication errors. The nurse–patient ratio is determined based on the medication service fee system, in which the Medical Care Act allows nursing administrators to assign nurses to wards with high workloads, depending on the daily situation. In other words, the exercise of leadership by nursing managers in the assignment of nurses can help devise ways to reduce medication errors [29]. Nurse managers should consider the number of nurses and their educational level, specific competencies and skills, and nursing-sensitive measures in settings with different patient characteristics [30].

Based on previous research, nursing-related policies, including employment, impact providing high-quality care and patient outcomes [31,32]. The unique nursing system in Japan, characterized by an employment dependent on management and a wide range of duties, is believed to affect patient outcomes, but this aspect has not been clarified.

The quality of nursing care affects patient safety and outcomes since nurses spend more time with patients than other healthcare providers. Missed nursing care may occur occasionally in all wards. The literature suggests that a positive patient safety culture in a non-punitive environment enhances voluntary reports of near-misses and errors [33]. Missed care was associated with falls, and patient safety culture dimensions explained up to 30% of the variance in missed nursing care, 26% of quality-of-care concerns, and 15% of vascular access device events [34]. The highest level of incomplete nursing care was associated with decreased patient safety factors linked with manager expectations and actions promoting safety, teamwork within and across hospital wards, feedback and communication about errors, and hospital handoff transitions [35].

Nurses often struggle to reduce medication errors. In particular, the incidence of medication errors is high, and 77% of medication near-miss/adverse events were caused by nurses in Japan [36]. According to a scoping review in 2018, with a median of 51.2% of all events, 34.3% and 83% were preventable [37]. As in the results of this study, medication errors occur more frequently in wards where invasive procedures and narcotics are used more frequently, and this result is consistent with the potential generalizability worldwide. Recent studies focused on artificial intelligence (AI) or computerized technologies to reduce medication errors using computerized physician order entry, automated drug distribution systems, AI-based prototype intravenous poles, and vital sign data within an AI framework [38]. Technologies may help reduce medication errors; hence, the most commonly identified issues related to medication incidents were related to digital communication [39,40,41].

This study was conducted based on the hypothesis that the risk of adverse events may increase with an increase in ward activity for the same patient profile. The most commonly reported adverse events are operative/surgical-related [36,37]. Regarding medication errors, an increase in the number of anesthesia (injections) and invasive procedures impacted adverse events, suggesting that increased opportunities for errors may have influenced adverse events. However, an increase in the number of patients undergoing surgery affected the suppression of events. This may be because patients who underwent surgery had fewer opportunities to make mistakes, considering their absence from the ward during the surgery.

### Limitations

This study has some limitations. First, data on nurse staffing hours in the analysis were obtained only during work hours without overtime. Future analysis should consider overtime hours. In addition, the working hours of nursing assistants also affect the quality (content) of nursing time per patient; however, this was not considered due to limitations in data acquisition. Furthermore, the analysis was conducted on a ward-day basis to examine ward risk factors. However, since patient risk and ward risk have complex influences, the patient basis should be examined by adding ward variables. This study targeted facilities that calculate the 1:7 acute stage basic inpatient hospitalization charge, and nurse staffing is provided within the scope of the facility standards; therefore, the generalization of the results would be difficult. Since not all medical institutions have an abundance of nurses daily, a critical point of nurse staffing may have affected the results. Although hospitals significantly impact adverse events, differences in patient demographics are another factor. This study considered the number of incident reports as the outcome. Therefore, the reporting status of medical facilities may have been highly dependent on the reporting status of the hospital, which may have resulted in hospital differences in adverse event occurrences. However, the discrepancy between the results of our study and those of previous studies in the differences between day and night shifts is probably due to some factors that we have not captured, and these factors are probably caused by the Japanese healthcare policy and the characteristics of nurses’ work. We believe that clarifying this point is the subject of our future research.

## 5. Conclusions

The medication errors caused by nurses were influenced by nursing time in opposite trends during day and night shifts. The results revealed that increasing the nursing time during day shifts would not prevent medication errors. They suggest that an increase in nursing time may indicate an increase in workload. In cases like Japan, where nurses have a wide range of duties, assessing the workload, nurse-to-patient ratio, and nursing time is necessary to improve patient safety.

Policymakers must establish a system that continuously monitors nursing practices and patient outcomes to improve quality. This system should include evaluating Japan-specific duties and workload assessments to effectively assess patient outcomes.

## Figures and Tables

**Table 1:** Characteristics of the hospitals.

Hospital ID	Number of Wards	Total Number of Patients	Age		Sex: Male		Surgical Patient		Nursing Time per Patient			
									Day Shift (8 h)		Night Shift (16 h)	
			Mean	SD	Number	%	Number	%	Mean	SD	Mean	SD
Hospital 1	5	4249	73.11	14.86	2179	54.9	2114	53.2	1.96	0.61	1.38	0.19
Hospital 2	9	8438	72.87	15.97	4386	54.5	4580	56.9	1.98	0.53	1.59	0.19
Hospital 3	8	6253	71.39	14.93	3169	53.4	3564	60.1	1.99	0.52	1.47	0.27
Hospital 4	6	5883	70.96	15.85	2842	51.0	3044	54.6	2.08	0.53	1.55	0.23
Hospital 5	5	3823	73.57	14.33	2044	57.0	1780	49.7	2.08	0.60	1.45	0.23
Hospital 6	9	7014	70.57	15.28	3826	57.3	3599	53.9	1.95	0.47	1.49	0.19
Hospital 7	7	6318	70.00	15.66	3313	56.9	3073	52.8	2.42	0.60	1.62	0.25
Hospital 8	8	6305	72.81	14.14	3330	56.5	3250	55.1	2.01	0.48	1.26	0.17
Hospital 9	12	9642	69.74	14.39	4864	52.2	4811	51.6	1.37	0.20	1.39	0.21
Hospital 10	8	6262	70.65	14.76	3435	56.1	3565	58.3	2.27	0.54	1.49	0.26
Total	77	64,187	71.43	15.08	33,388	54.8	33,380	54.8	1.96	0.58	1.46	0.24

**Table 2:** Comparison between medication error and non-medication error based on ward characteristics (N = 27,629 ward days).

	Non-Medication Error Group N = 23,840		Medication Error Group N = 3789		*p* *
	Mean	SD	Mean	SD	
**Nursing time per patient**					
Day shift (8 h), hours	1.95	0.58	2.06	0.58	<0.01
Night shift (16 h), hours	1.46	0.24	1.46	0.24	0.828
**Patients’ background**					
65 years old and over, %	71.96	74.14	73.17	75.00	<0.01
Men, %	57.67	58.33	57.96	58.49	0.64
Discharge, %	6.87	6.25	7.12	6.45	0.01
Admission, %	5.99	4.88	6.20	5.36	<0.01
Hospital death, %	0.19	0.00	0.19	0.00	0.58
Sedative hypotics, %	13.03	12.00	13.29	12.50	<0.01
Psychotropic, %	9.73	8.33	10.44	9.09	<0.01
Hypertention, %	33.72	31.25	33.34	30.61	0.09
Osteoporosis, %	4.89	3.45	4.86	3.64	0.22
Anemia, %	14.67	12.77	13.96	12.07	<0.01
CCI score 1, %	23.82	23.26	24.05	23.53	0.13
CCI score 2, %	15.83	15.38	15.81	15.38	0.70
CCI score 3, %	10.85	10.00	10.42	9.62	<0.01
CCI score 4 or more, %	8.03	6.52	7.56	6.25	<0.01
Emergency hospitalization, %	0.93	0.00	0.92	0.00	0.91
Surgery, %	1.41	0.00	1.46	0.00	0.18
Injection, %	26.77	26.00	27.98	26.83	<0.01

* continuous variable: Mann–Whitney U test, discrete variable: χ2 test.

**Table 3:** Comparison between medication error and non-medication error groups from the perspective of SCNMN (N = 27,629 ward days).

	Non-Medication Error Group N = 23,840		Medication Error Group N = 3789		*p* *
	Mean	SD	Mean	SD	
**≪Monitoring and treatment** **≫**					
Wound treatment (excluding treatment of pressure ulcer), %	4.72	2.33	4.60	2.27	0.05
Treatment of pressure ulcers, %	0.28	0.00	0.29	0.00	0.40
Respiratory care (except for only sputum aspiration), %	9.00	6.90	9.46	7.50	<0.01
Management of three or more intravenous lines at the same time, %	5.91	3.70	6.07	3.77	0.42
ECG monitor management, %	19.71	15.69	19.59	15.69	0.93
Syringe driver management, %	2.11	0.00	2.40	0.00	<0.01
Management of blood transfusion and blood product, %	1.86	0.00	2.00	0.00	0.03
Professional treatment, %	25.91	23.53	25.98	23.53	0.72
Use of antineoplastic agents (injection only), %	2.02	0.00	2.18	0.00	0.08
Management of oral administration of antineoplastic agents, %	1.70	0.00	1.67	0.00	0.51
Use of narcotics (injection only), %	2.05	0.00	2.20	0.00	0.07
Internal use of narcotics, application, management of suppositories, %	1.67	0.00	1.63	0.00	0.86
Radiation therapy, %	1.52	0.00	1.64	0.00	<0.01
Immunosuppressant management, %	9.45	6.67	9.28	6.82	0.28
Use of pressor agent (injection only), %	1.59	0.00	1.60	0.00	0.34
Use of antiarrhythmic agent (injection only), %	0.36	0.00	0.38	0.00	0.73
Use of continuous infusion of antithrombotic embolic drug, %	3.09	2.08	3.10	2.08	0.72
Drainage management, %	5.86	3.03	5.63	2.63	<0.01
Treatment in a sterile treatment room, %	1.51	0.00	1.64	0.00	0.25
**≪Patients’ functional state** **≫**					
Turnover (Partly assisted), %	24.89	21.43	26.61	22.22	0.03
Turnover (Fully assisted), %	16.64	14.89	16.52	14.89	0.94
Transfer (Partly assisted), %	30.36	29.17	30.34	28.95	0.81
Transfer (Fully assisted), %	12.24	10.34	12.33	10.34	0.98
Oral care, %		43.18	45.93	43.75	0.07
Meal intake (Partly assisted), %	25.75	24.07	25.42	24.39	0.52
Meal intake (Fully assisted), %	10.55	7.89	10.39	8.16	0.03
Personal dressing (Partly assisted), %	27.08	26.67	26.22	25.81	0.04
Personal dressing (Fully assisted), %	21.69	19.15	21.76	19.57	0.11
No able to receive directions on medical care and treatment, %	19.87	13.33	20.42	13.73	0.04
Engaged in dangerous behavior, %	9.62	6.82	9.45	7.14	0.09
**≪Surgery and emergency care** **≫**					
Craniotomy (within 7 days from the day of surgery), %	0.13	0.00	0.12	0.00	0.28
Thoracotomy (within 7 days from the day of surgery), %	0.10	0.00	0.11	0.00	0.69
Laparotomy (within 4 days from the day of surgery), %	0.50	0.00	0.53	0.00	0.38
Bone surgery (within 5 days from the day of surgery), %	1.60	0.00	1.73	0.00	0.59
Thoracoscopic/laparoscopic surgery (within 3 days from the day of surgery), %	1.02	0.00	0.97	0.00	0.05
General anesthesia/spinal anesthesia surgery (within 2 days from the day of surgery), %	2.88	0.00	3.03	0.00	0.22
Percutaneous endovascular treatment, %	0.37	0.00	0.47	0.00	<0.01
Treatment such as percutaneous myocardial ablation, %	0.33	0.00	0.41	0.00	<0.01
Invasive gastrointestinal treatment, %	0.63	0.00	0.80	0.00	<0.01
Eye surgery, %	0.14	0.00	0.18	0.00	<0.01

* continuous variable: Mann–Whitney U test, discrete variable: χ2 test.

**Table 4:** Logistic regression analysis for the variables related to medication errors (N = 27,629 ward days).

	β	Odds	Odds 95% CI※1		*p*
			Lower	Upper	
Nursing time per patient: day shift (8 h), hour	0.27	1.31	1.21	1.42	<0.01
Nursing time per patient: night shift (16 h), hour	−0.59	0.55	0.46	0.67	<0.01
Use of narcotics (injection only), %	0.05	1.05	1.03	1.07	<0.01
Percutaneous endovascular treatment, %	0.03	1.03	1.01	1.06	<0.01
Treatment such as percutaneous myocardial ablation, %	0.04	1.04	1.02	1.06	<0.01
Invasive gastrointestinal treatment, %	0.03	1.03	1.02	1.05	<0.01
Oral care, %	0.00	1.00	1.00	1.01	<0.01
Surgery, %	−0.04	0.96	0.94	0.98	<0.01

※1 CI; confidence interval; Note1 Nagelkerke R2 = 0.03, Hosmer—Lemeshow test *p* = 0.50; Note2 Adjusted for the number of patients in hospital (%), hospital nest.

## Data Availability

The datasets generated and/or analyzed in the current study are not publicly available because of contracts with hospitals providing data to the database. The data are available from the coauthor (H.H.) upon reasonable request.

## Supplementary Materials

The following supporting information can be downloaded at: https://www.mdpi.com/article/10.3390/nursrep15010012/s1, Table S1: Number of medical errors in the hospital wards. Table S2: Number of patients by hospital ward day.

## Public Involvement Statement

No public involvement in any aspect of this research.

## Guidelines and Standards Statement

This manuscript was drafted against The Strengthening the Reporting of Observational Studies in Epidemiology (STROBE) Statement for Observational studies research.
