# Supplementary material for: Influence of Nursing Time and Staffing on Medication Errors: A Cross-Sectional Analysis of Administrative Data
Source: Nurs Rep. 2025 Jan 5;15(1):12. doi: 10.3390/nursrep15010012 (PMC11767928; doi:10.3390/nursrep15010012)
Supplement: Supplementary file 1 [file nursrep-15-00012-s001.zip › nursrep-3303547-supplementary.pdf]

Supplementary Table S1. Number of medical errors in the hospital wards.

Hospital ID	Non-medication errors group		Medication errors group		Total of events	
	Number of ward-days	%	Number of ward-days	%	Number of ward-days	%
Hospital 1	1,689	92.50	137	7.50	1,826	100.00
Hospital 2	2,846	86.93	428	13.07	3,274	100.00
Hospital 3	2,564	89.18	311	10.82	2,875	100.00
Hospital 4	1,867	86.52	291	13.48	2,158	100.00
Hospital 5	1,510	83.61	296	16.39	1,806	100.00
Hospital 6	2,739	83.99	522	16.01	3,261	100.00
Hospital 7	1,858	78.93	496	21.07	2,354	100.00
Hospital 8	2,445	83.56	481	16.44	2,926	100.00
Hospital 9	3,917	90.44	414	9.56	4,331	100.00
Hospital 10	2,405	85.34	413	14.66	2,818	100.00
Total	23,840	86.29	3,789	13.71	27,629	100.00

Supplementary Table S2. Number of patients by hospital ward day.

Hospital ID	Number of ward days	Number of patients	
		mean	SD
Hospital 1	1,826	41.8	5.3
Hospital 2	3,274	42.3	4.9
Hospital 3	2,875	37.8	6.0
Hospital 4	2,158	43.5	5.7
Hospital 5	1,806	37.9	5.6
Hospital 6	3,261	39.5	4.4
Hospital 7	2,354	40.9	6.3
Hospital 8	2,926	49.8	3.9
Hospital 9	4,331	41.4	5.5
Hospital 10	2,818	39.8	7.0
Total	27,629	41.6	6.4
